# The dynamic impact of higher education on youth employment opportunities and quality: an empirical analysis based on age-period-cohort models

**DOI:** 10.3389/fsoc.2025.1680077

**Published:** 2025-10-29

**Authors:** Longyin Chen, Jiru Guo, Wenting Li, Guyue Wang

**Affiliations:** ^1^Graduate School, Xi'an Physical Education University, Xi'an, China; ^2^Country School of Economics and Management, Shanghai University of Sport, Shanghai, China; ^3^School of Media and Art, Tianjin University of Sport, Tianjin, China; ^4^School of Physical Education, Xi'an Physical Education University, Xi'an, China

**Keywords:** higher education, employment opportunities, employment quality, dynamic trends, HAPC, CSS

## Abstract

**Introduction:**

Education serves as a crucial mechanism influencing individuals’ labor income and social mobility in modern society, and whether one has attained higher education has become a key determinant in the allocation of competitive opportunities within the labor market. However, the impact of this determinant on youth employment is inherently dynamic, shaped both by micro-level life course trajectories and by macro-level processes of social transformation.

**Methods:**

Drawing on eight waves of data from the China Social Survey (CSS) covering 2006–2021, this study applies a Hierarchical Age–Period–Cohort Cross-Classified Random Effects Model (HAPC-CCREM) to assess how higher education influences young people’s employment outcomes in the labor market. The analysis considers two dimensions of employment performance—opportunities and quality—while also mapping dynamic trends across age, period, and cohort.

**Results:**

Higher education has a significant impact on all three dimensions of youth employment. Age: For higher-education youth, both employment opportunities and quality follow an inverted U-shaped curve, whereas for non-higher-education youth both indicators increase gradually. Period: Employment opportunities for both groups have undergone cyclical declines since 2006, but the decrease has been less steep for higher-education graduates, who have consistently outperformed their non-higher-education peers. In terms of employment quality, higher-education graduates show a fluctuating upward trend, while non-graduates maintain relatively stable levels. Cohort: Among higher-education cohorts, the effect on both employment opportunities and quality displays a “gradual rise–sharp decline” trajectory, whereas among non-higher-education cohorts, the pattern is one of “moderate fluctuations–mild improvement.”

**Conclusion:**

While higher education significantly enhances youth employment opportunities and job quality, its returns decline in the later stages across all three temporal dimensions and are increasingly constrained by credential inflation and labor market structural transformations. This underscores the urgency of aligning higher education with the evolving labor market and implementing targeted employment policies.

## 1 Introduction

The relationship between higher education and labor market outcomes has been extensively explored in social science research, particularly regarding occupational attainment and wage premiums in developed nations (Carlsen et al., 2016; Hill, 2016; Bukodi and Goldthorpe, 2011). In contrast, for developing countries, the benefits of education are often reflected in enhanced employment prospects and improved employment quality, including factors such as working hours and job security (Ukaj et al., 2024). As of 2023, China has established one of the world’s largest tertiary education systems, with a gross enrollment rate of 60.20% (GOV, 2024) and 11.58 million college graduates. However, by June 2023, the unemployment rate among urban youth aged 16–24 had reached 21.3%, with an average weekly workload of 48.7 h (NBS). This suggests that in China, the interplay between education and the labor market is particularly intricate.

In examining the relationship between higher education and the labor market, two foundational theories dominate scholarly debate. The first, educational functionalism, argues that higher education strengthens workers’ skills and improves the efficiency of occupational matching through the accumulation of human capital and the operation of selection mechanisms (Parsons, 1961; Arrow, 1973). These processes, in turn, stimulate productivity growth and expand income levels. Consequently, greater investment in education corresponds to the institutional demand for human capital in industrialized societies, exerting a positive influence on the employment prospects of highly educated individuals. The second, job competition theory, contends that the signaling value of education hinges on its scarcity. Thurrow’s (1975) job competition model emphasizes that employment opportunities arise from the dynamic matching of an individual’s relative educational ranking within the competitive queue and the structural demands of available jobs. As higher education expands, employers respond by raising educational thresholds, a phenomenon Collins (2019) refers to as “diploma inflation.” While individuals’ absolute education levels rise, their relative advantage diminishes, intensifying competition for high-end jobs among the highly educated. This, in turn, contributes to structural unemployment and a decline in employment quality. In recent years, a new wave of technological transformation—driven by informatization, digitization, and automation—has profoundly reshaped the demand structure for human capital in the labor market. This shift manifests in two main ways. On the one hand, advances in technology have markedly increased the productivity of highly skilled occupations. On the other, they have gradually displaced many medium- and low-skill jobs, fueling a polarized employment structure. For example, Campos-González and Balcombe (2024), applying the “Race between Education and Technology” framework to the case of Chile, found that when the expansion of an education system fails to keep pace with the growing skill requirements generated by technological change, the wage premium for highly skilled workers continues to widen. Such wage polarization, in turn, amplifies income inequality within the labor market.

These theories each have their own emphasis in explaining the relationship between higher education and employment, and they also provide an important theoretical foundation for this study. Therefore, considering the current technological advancements and the macro-structural changes brought about by globalization, as well as the ongoing adjustments in the labor market, this study further expands its research perspective by focusing on both employment opportunities and employment quality. It systematically examines the dynamic changes in the returns to higher education within the age-period-cohort triple framework. This paper aims to answer the following questions: 1. In contemporary China, does higher education still provide workers with significant advantages in employment opportunities and improvements in employment quality? 2. How do these returns exhibit changing trends across different age groups, different socio-economic periods, and different birth cohorts? To this end, the study employs data from eight waves (2006–2021) of the Chinese Social Survey (CSS) to construct a Hierarchical Age-Period-Cohort Cross-Classified Random-Effects Model (HAPC), systematically analyzing the dynamic impact of higher education on youth employment opportunities and quality, as well as its generational differences. This not only helps to understand the underlying causes of employment challenges faced by Chinese youth but also provides valuable insights for improving the structure of higher education, optimizing human capital supply, and supporting the government in formulating precise and effective employment policies.

The study employs eight waves of data (2006–2021) from the China Social Survey (CSS) to construct a Hierarchical Age-Period-Cohort (HAPC) random effects model. This approach enables a systematic assessment of how higher education influences youth employment opportunities and job quality, while also identifying generational disparities. The findings aim to enhance our understanding of the structural roots of youth employment challenges in China, inform the optimization of higher education systems, improve the alignment of human capital supply with labor market demands, and guide policymakers in designing targeted, evidence-based employment strategies.

## 2 Literature review

Research findings on the relationship between higher education and the labor market are abundant. Scholars have examined the role of education in shaping workers’ employment opportunities, income inequality, occupational status, and job stability through diverse perspectives, historical data, and methodologies (Hendel et al., 2005; Chen and Wu, 2007; Mirowsky, 2017). Theoretically, early human capital theories emphasized that education enhances workers’ market competitiveness and economic returns by improving skills and productivity (Burgess, 2016). Signaling theory, on the other hand, posits that education serves as a credential signaling ability and potential to employers during job searches (Kroch and Sjoblom, 1994). Subsequently, skill-biased technological change theory and global value chain division of labor theory further revealed how educational attainment interacts with macroeconomic forces to jointly shape differentiated labor demand structures (Autor, 2005; Lauder et al., 2018). Empirical studies consistently find that higher education is significantly associated with higher wages, entry into the formal sector, and access to quality job opportunities (Bar-Haim et al., 2019), but its rate of return exhibits significant heterogeneity across different contexts. First, variations exist between countries and regions. Higher education returns in developed economies are more stable and structured, enabling long-term accumulation of income and career advantages. In some developing countries, however, limited labor market absorption capacity leads to fluctuating or even declining educational premiums. Second, industrial structure and technological change profoundly influence educational returns. As the Race between Education and Technology framework indicates, skill-biased technological change continuously increases the relative demand for highly skilled labor, thereby boosting educational returns, though this effect is partially offset by expanding educational supply (Goldin and Katz, 2007). Finally, individual and societal contexts create heterogeneity. Factors such as gender, family socioeconomic status, and field of study all influence variations in the income premium and employment quality associated with higher education (Guo et al., 2025).

This disparity highlights the need for country-specific research on the relationship between education and the labor market, with full consideration of macroeconomic conditions, historical contexts, and individual life cycles. The Hierarchical Age–Period–Cohort (HAPC) model offers a robust analytical framework, widely adopted in disciplines such as health sociology, epidemiology, and educational sociology for its ability to disentangle the independent effects of age, period, and cohort factors. For example, Bar-Haim et al. (2019) applied the Age–Period–Cohort (APC) model to show that the rise in overeducation among European youth was largely driven by period effects, such as economic crises reducing job quality; Vera-Toscano and Meroni (2021) identified cohort effects reflecting intensified intergenerational competition within expanded higher education cohorts. Despite its advantages, the application of the HAPC framework in developing and middle-income countries remains scarce. Existing studies often rely on cross-sectional data and examine only a single employment outcome, limiting their ability to uncover systematic differences in educational returns across age, period, and cohort dimensions. In the Chinese context, there is a particular lack of large-scale empirical research that simultaneously evaluates employment opportunities and job quality. To address this gap, the present study draws on Chinese Social Survey (CSS) data and applies the HAPC model to systematically assess how higher education influences youth employment opportunities and job quality across age, period, and cohort dimensions, providing both empirical evidence and theoretical foundations for subsequent policy interventions.

### 2.1 Age effect of higher education on youth employment

The age effect captures how age-related changes in physical capacity, social roles, and accumulated experience influence individuals’ behaviors and perceptions. This effect typically follows a non-linear “inverted U-shaped” trajectory, where human capital accumulation initially strengthens competitiveness but later declines due to skill obsolescence and health deterioration. Regarding employment quality, the influence of age operates through a more intricate mechanism. Traditional human capital theory posits that, with age, workers accumulate experience and skills that enhance their human capital, thereby increasing economic returns and improving employment quality. However, prolonged tenure in a single occupation is often associated with lower self-assessed job evaluations and declining job satisfaction among older workers. These trends may be partly attributable to heightened family-related financial pressures during this life stage, including expenses related to marriage, child-rearing, and housing.

Similarly, research on the relationship between age and the employment outcomes of higher education graduates has produced mixed results. For example, Wang (2025), using data from the China Family Panel Studies (CFPS) covering 2016–2022, found that the employment rate among young people with higher education exhibited a downward trend. Li and Wu (2025) indicate that highly educated workers follow an inverted U-shaped employment trajectory, enjoying broader career choices during the “golden period” between ages 25 and 45. In terms of employment quality, Topel highlights that college graduates gain early career advantages over high school graduates, including better access to high-paying industries and more significant job roles. These benefits accumulate over time, further widening the earnings gap (Topel, 1991). Similarly, in a study on Hong Kong, China, DiPrete and Eirich (2006) observe that the returns to higher education tend to increase with age. However, research focusing specifically on mainland Chinese workers remains limited.

### 2.2 Period effect of higher education on youth employment

Period effects refer to immediate changes in the external environment, such as historical events, environmental shifts, or technological innovations. Worldwide, youth labor force participation rates have generally shown a declining trend in recent years (International Labour Organization, 2020). China has also exhibited a similar trend, with macroeconomic data showing that the youth labor force participation rate (ages 25–44) decreased from 92.70% in 2000 to 85.72% in 2015, a drop of 6.98% (National Bureau of Statistics, 2016). However, related research indicates that China’s youth employment quality composite index has increased from 0.56 to 0.64 (Guo et al., 2022). The core reason for this apparent contradiction lies in the dual impact of technological advancements and industrial restructuring. Traditional manufacturing jobs have been diminishing due to automation and shifts in globalization, reducing employment opportunities for low-skilled youth. Conversely, the growth of the digital economy and the service sector has increased demand for highly skilled labor, resulting in improvements in quality indicators such as wages and job stability. This indirectly highlights the significant role of higher education in the labor market.

The employment status of higher education graduates results from a combination of factors, including social development, economic transformation, national policies, and demographic changes. Specifically, since 2005, the share of China’s tertiary sector has exceeded 50%, driving a surge in demand for highly skilled workers (Tanshu, 2025). However, there was a mismatch between the structure of higher education fields and market needs during this period, with high demand for engineering graduates but challenges for liberal arts graduates to secure jobs with appropriate salaries and qualifications (Yang et al., 2015). After 2016, the rise of new industries in China, including the digital and platform economies, reshaped the job market, with flexible work and skill-based demand becoming increasingly important (Liu et al., 2022). The technological revolution has accelerated the transition to a knowledge-based economy, leading to a significant increase in high-quality jobs in STEM (science, technology, engineering, and mathematics) fields and heightened demand for highly educated workers (Makgato, 2019). This shift has helped alleviate labor market overcrowding and improved job quality in some sectors (Fang, 2021). However, the COVID-19 pandemic, which emerged at the end of 2019, caused significant disruption to social activities and the labor market. University graduates faced exceptionally tough employment conditions, with higher employment thresholds and increasing job market competition. As a result, it can be predicted that the returns to higher education were significantly lower during these 2 years. Overall, in the context of China’s unique social transformation, further research using updated and more comprehensive data is needed to explore the evolving trends in period effects of returns to higher education.

### 2.3 Cohort effect of higher education on youth employment

Cohort effects refer to the shared characteristics of individuals born during the same period and their tendency to evolve over time as they experience specific historical and social contexts (Yang and Land, 2013). The economic systems and educational policies unique to each era significantly influence the returns to higher education for each generation. From the founding of the People’s Republic of China to the reform and opening up, China operated under a planned economy with a “uniform recruitment and uniform distribution” employment system, leading to a lifelong employment model. Unemployment risks were low, but career choices were limited, and the primary labor force consisted of the “post-50s” and earlier cohorts. During the Cultural Revolution, when the belief that “studying is useless” emerged, education was temporarily halted, resulting in a severe employment crisis and a surge in youth unemployment. In 1977, the resumption of the national college entrance exams allowed young people to secure jobs through higher education. Following this, the labor market entered a stage of preparation for reform, and the government introduced the “three-integrated” employment policy, which combined labor department recruitment, voluntary organization, and self-employment. This enabled the “post-70s” and “post-80s” cohorts to find jobs and improve employment quality (Wei et al., 2024). Consequently, employment opportunities and quality for these groups saw an upward trajectory. It wasn’t until the late 1990s that China expanded higher education to provide more individuals with opportunities for upward mobility. However, this expansion led to the depreciation of academic qualifications and the emergence of “over-education,” which impacted the “post-90s” and “post-00s” (Pei et al., 2023). According to Wang (2025), an analysis of youth employment from 2016 to 2022 revealed rising weekly working hours, slower income growth, and deteriorating employment quality among younger cohorts. Furthermore, it is difficult to draw comparisons beyond a single cohort (5 years), as graduates from the expanded higher education system primarily compete within the same cohort and with previous graduates. Thus, we argue that the negative impact of higher education expansion on youth employment is largely a cohort effect, which should be carefully examined through cohort comparisons.

## 3 Data and methods

### 3.1 Data

The data used in this study were derived from the Chinese Social Survey (CSS) conducted in 2006, 2008, 2011, 2013, 2015, 2017, 2019, and 2021, comprising a total of eight surveys. The selection of CSS data is based on several factors: firstly, CSS is a long-term national survey that focuses on labor and employment, family life, social attitudes, and societal changes in China, aligning with the core theme of this study, which is the examination of social change in employment; secondly, the data span a sufficient time period; and thirdly, the survey items remain largely consistent across the years. After combining data from these eight waves, the study addressed age discrepancies (focusing on individuals aged 18–44), removed samples with missing employment opportunity data, eliminated cases with fewer than three of the four employment quality dimensions, and excluded individuals with missing educational background or control variables. Ultimately, 27,958 valid samples were retained for analysis.

### 3.2 Variables

#### 3.2.1 Dependent variables

##### 3.2.1.1 Employment opportunities

Employment opportunities are assessed through the question, “What is your current job situation?” A value of 1 is assigned to individuals with a job, while a value of 0 is assigned to those without employment.

##### 3.2.1.2 Employment quality

Based on the selection of multidimensional employment quality indicators by both domestic and international scholars, and considering the available CSS data, the employment quality index is constructed using four dimensions: wage level, job intensity, job stability, and job security (Erhel et al., 2014; Zhao, 2016; Li and Yuan, 2017). These dimensions are measured by the following indicators: average monthly income, weekly working hours, whether an employment contract has been signed, and whether social security benefits such as old-age pension, healthcare, unemployment insurance, work-related injury insurance, and maternity benefits are provided (Guo et al., 2022).

The employment quality index was calculated as follows: in the first step, the four dimensions were standardised, see Equations 1, 2:

Positive normalization formula:


(1) $$X_{\mathrm{ij}}^{\mathrm{nor}}=\frac{X_{\mathrm{ij}}-\min_{j}}{\max_{j}-\min_{j}}$$

Negative normalization formula:


(2) $$X_{\mathrm{ij}}^{\mathrm{nor}}=\frac{\max_{j}-X_{\mathrm{ij}}}{\max_{j}-\min_{j}}$$

Where $X_{\mathrm{ij}}^{\mathrm{nor}}$ is the standard indicator, i denotes the number of samples, j = {1, 2, 3, 4} denotes each of the four dimensions of employment quality, $\max_{j}$ and $\min_{j}$ denote the maximum and minimum values of indicator j, respectively.

In the second step, the employment equality was calculated using the equal-all average method based on the standardization of the indicators for the four dimensions, see Equation 3:


(3) $$Q_{i}=\frac{1}{4}\Sigma_{j=1}^{4}X_{\mathrm{ij}}^{\mathrm{nor}}$$

The final calculation results in an employment quality index of 0–1, with higher values being associated with better employment quality.

#### 3.2.2 Independent variables

##### 3.2.2.1 Higher education

Re-code the question “What is the highest level of education you have ever attended (including the one you are currently attending)?” in the CSS. The question was re-coded so that the highest level of education was 0 for those who had attended school, primary school, junior high school, high school, secondary school, vocational high school, or technical school, indicating that they had not received higher education; and 1 for those whose highest level of education was college, undergraduate, or graduate school, indicating that they had received higher education.

##### 3.2.2.2 Age

Age was limited to 18–44 years old and age squared was calculated.

##### 3.2.2.3 Period

The period is the corresponding year of the CSS data survey.

##### 3.2.2.4 Cohort

The birth cohorts span from 1962 to 2003, divided into 9 groups at 5-year intervals, aligning with the Chinese social convention of naming generations by the decade endings of “0” and “5” (e.g., “post-60s,” “post-70s,” “post-95 s,” etc.). The specific groupings are: 1962–1965, 1966–1970, 1971–1975, 1976–1980, 1981–1985, 1986–1990, 1991–1995, 1996–2000, and 2001–2003.

#### 3.2.3 Control variables

This paper introduces gender, marriage, household registration, political profile and geographical variables, and the detailed coding is shown in Table 1. Among them, according to the National Bureau of Statistics, the eastern, central and western regions are divided.

**Table 1:** Descriptive statistics of basic variables.

Variable	Mean ± standard deviation/Percentage
Employment quality	0.36 ± 0.23
Employment opportunities	
Non-work = 0	25.25%
Work = 1	74.75%
Higher education	
Non-higher education = 0	71.69%
Higher education = 1	28.31%
Age	32.91 ± 7.62
Gender	
Female = 0	53.84%
Male = 1	46.16%
Marital status	
Unmarried = 0	25.61%
Married = 1	74.39%
Household registration	
Rural = 0	62.82%
Non-farmer = 1	30.26%
Urban = 2	6.92%
Political affiliation	
Non-Communist Party of China = 0	91.17%
Chinese Communist Party members = 1	8.83%
Region	
Eastern = 0	47.12%
Central = 1	26.78%
Western = 2	26.10%
Period	
2006	8.55%
2008	6.87%
2011	9.98%
2013	16.26%
2015	14.92%
2017	14.44%
2019	14.45%
2021	14.53%
Cohort	
1962–1965	2.69%
1966–1970	8.62%
1971–1975	17.59%
1976–1980	18.10%
1981–1985	16.82%
1986–1990	16.48%
1991–1995	10.65%
1996–2000	7.16%
2001–2003	1.88%

### 3.3 Research methodology

This study employs the APC model, commonly used in social science research to analyze trends and changes, to explore the age, period, and cohort effects of higher education on employment. However, the traditional APC model faces a challenge: since “cohort = period-age,” there is covariance between these components, making it difficult to independently decompose them. As a result, traditional regression methods cannot provide a unique solution for the parameters. In 2006, Yang and Land introduced the Hierarchical APC-cross-classified random effects Models, which addresses this covariance issue by incorporating the concept of stratification. The principle of this model is to decompose the effects of age, period, and cohort on the dependent variable across different levels. It assumes that the fixed effects at the individual level and the random effects at the cohort level can solve the problem. This approach offers a novel perspective for APC analysis. Consequently, this study tests the proposed hypotheses using the HAPC model. Referring to relevant studies (Zhang and Wang, 2024), the formula for the HAPC model on the impact of higher education on employment is presented as follows:

#### 3.3.1 Model 1: Baseline model

##### 3.3.1.1 Level 1

(4) $$\begin{array}{c} Y_{\mathrm{ijk}}=\beta_{0\mathrm{jk}}+\beta_{1}\mathrm{ED}U_{\mathrm{ijk}}+\beta_{2}\mathrm{AG}E_{\mathrm{ijk}}+\beta_{3}\mathrm{AG}E_{\mathrm{ijk}}^{2}+\\ \beta_{4}\mathrm{CO}N_{\mathrm{ijk}}+e_{\mathrm{ijk}},e_{\mathrm{ijk}}\sim N(0,\delta^{2}) \end{array}$$

Where $Y_{\mathrm{ijk}}$ represents the employment status level of individual i in period k and generation j; $\beta_{0\mathrm{jk}}$ is the intercept indicating the average employment score for the reference group at the mean age interviewed within a specific period and cohort. $\mathrm{ED}U_{\mathrm{ijk}}$ denotes the level of higher education; $\beta_{1}\;$ denotes the random coefficient of the level of higher education; $\mathrm{AG}E_{\mathrm{ijk}}$ and $\mathrm{AG}E_{\mathrm{ijk}}^{2}$ denote the age and age squared, respectively; $\beta_{2}$ and $\beta_{3}\;$ are fixed coefficients of age and age squared; $\mathrm{CO}N_{\mathrm{ijk}}$ denotes control variables; $\beta_{4}\;$ denotes fixed coefficients of control variables; and $e_{\mathrm{ijk}}$ is a random error term and follows a normal distribution. The same subscripts will not be repeated.

##### 3.3.1.2 Level 2

(5) $$\beta_{0\mathrm{jk}}=\pi_{0}+\mu_{0j}+v_{0k},\mu_{0j}\sim N(0,\sigma_{\mu }),\nu_{0k}\sim N(0,\sigma_{v})$$

Where $\pi_{0}$ is the intercept term, $\mu_{0j}$ is the cohort effect or residual random effect for cohort j, and $\nu_{0k}$ is the period effect or residual random effect for period k. And both $\mu_{0j}$ and $v_{0k}$ obey normal distributions.

Substituting Equation 5 into Equation 4 yields the fundamental Equation 6. The complete formula is as follows:


(6) $$\begin{array}{c} Y_{\mathrm{ijk}}=(\pi_{0}+\mu_{0j}+\nu_{0k})+\beta_{1}\mathrm{ED}U_{\mathrm{ijk}}+\beta_{2}\mathrm{AG}E_{\mathrm{ijk}}+\beta_{3}\mathrm{AG}E_{\mathrm{ijk}}^{2}+\\ \beta_{4}\mathrm{CO}N_{\mathrm{ijk}}+e_{\mathrm{ijk}},e_{\mathrm{ijk}}\sim N(0,\delta^{2}) \end{array}$$

#### 3.3.2 Model 2: Age effects

The full model of the age effect was obtained by adding the interaction terms of physical activity with age and age squared in the first level:


(7) $$\begin{array}{c} \begin{array}{l} Y_{\mathrm{ijk}}=(\pi_{0}+\mu_{0j}+\nu_{0k})+\beta_{1}\mathrm{ED}U_{\mathrm{ijk}}+\beta_{2}\mathrm{AG}E_{\mathrm{ijk}}+\\ \beta_{3}\mathrm{AG}E_{\mathrm{ijk}}^{2}+\beta_{4}\mathrm{AG}E_{\mathrm{ijk}}\mathrm{ED}U_{\mathrm{ijk}}+ \end{array}\\ \beta_{5}\mathrm{AG}E_{\mathrm{ijk}}^{2}\mathrm{ED}U_{\mathrm{ijk}}+\beta_{6}\mathrm{CO}N_{\mathrm{ijk}}+e_{\mathrm{ijk}},e_{\mathrm{ijk}}\sim N(0,\delta^{2}) \end{array}$$

#### 3.3.3 Model 3: Period effects

In order to estimate the period effect, following Cao et al. (2022), $\beta_{1k}$ in Equation 7 is replaced with $\beta_{1k}=\pi_{0}+\vartheta_{0k},\vartheta_{0k}\sim N(0,\sigma_{v})$ , resulting in the period effect model equation:


(8) $$\begin{array}{l} Y_{\mathrm{ijk}}=(\pi_{0}+\mu_{0j}+\nu_{0k})+(\pi_{0}+\vartheta_{0k})\mathrm{ED}U_{\mathrm{ijk}}\\ +\beta_{2}\mathrm{AG}E_{\mathrm{ijk}}+\beta_{3}\mathrm{AG}E_{\mathrm{ijk}}^{2}+\beta_{4}\mathrm{CO}N_{\mathrm{ijk}}\\ +e_{\mathrm{ijk}},e_{\mathrm{ijk}}\sim N(0,\delta^{2}) \end{array}$$

#### 3.3.4 Model 4: Cohort effects

Referring to Equation 8 and replacing $\beta_{1j}$ with $\beta_{1j}=\pi_{0}+\vartheta_{0j},\vartheta_{0j}\sim N(0,\sigma_{\mu })$ in Equation 7, the final formula obtained is Equation 9, which is the cohort effect model.


(9) $$\begin{array}{c} \begin{array}{l} Y_{\mathrm{ijk}}=(\pi_{0}+\mu_{0j}+\nu_{0k})+(\pi_{0}+\mu_{0j})\mathrm{ED}U_{\mathrm{ijk}}+\beta_{2}\mathrm{AG}E_{\mathrm{ijk}}\\ +\beta_{3}\mathrm{AG}E_{\mathrm{ijk}}^{2}+\beta_{4}\mathrm{CO}N_{\mathrm{ijk}}+e_{\mathrm{ijk}},e_{\mathrm{ijk}}\sim N(0,\delta^{2}) \end{array} \end{array}$$

## 4 Results

### 4.1 Descriptive statistics

The sample (N = 27,958) had a mean employment quality score of 0.36, with 74.75% employed. Most (71.69%) had no higher education, and the average age was 32.91 years. Females comprised 53.84% of the sample, 74.39% were married, and 62.82% were from rural areas. The majority (91.17%) were non-CPC members, and 47.12, 26.78 and 26.10% were from the eastern, central, and western regions, respectively (Table 1).

### 4.2 Changes in China’s youth employment status

**Figure 1:**
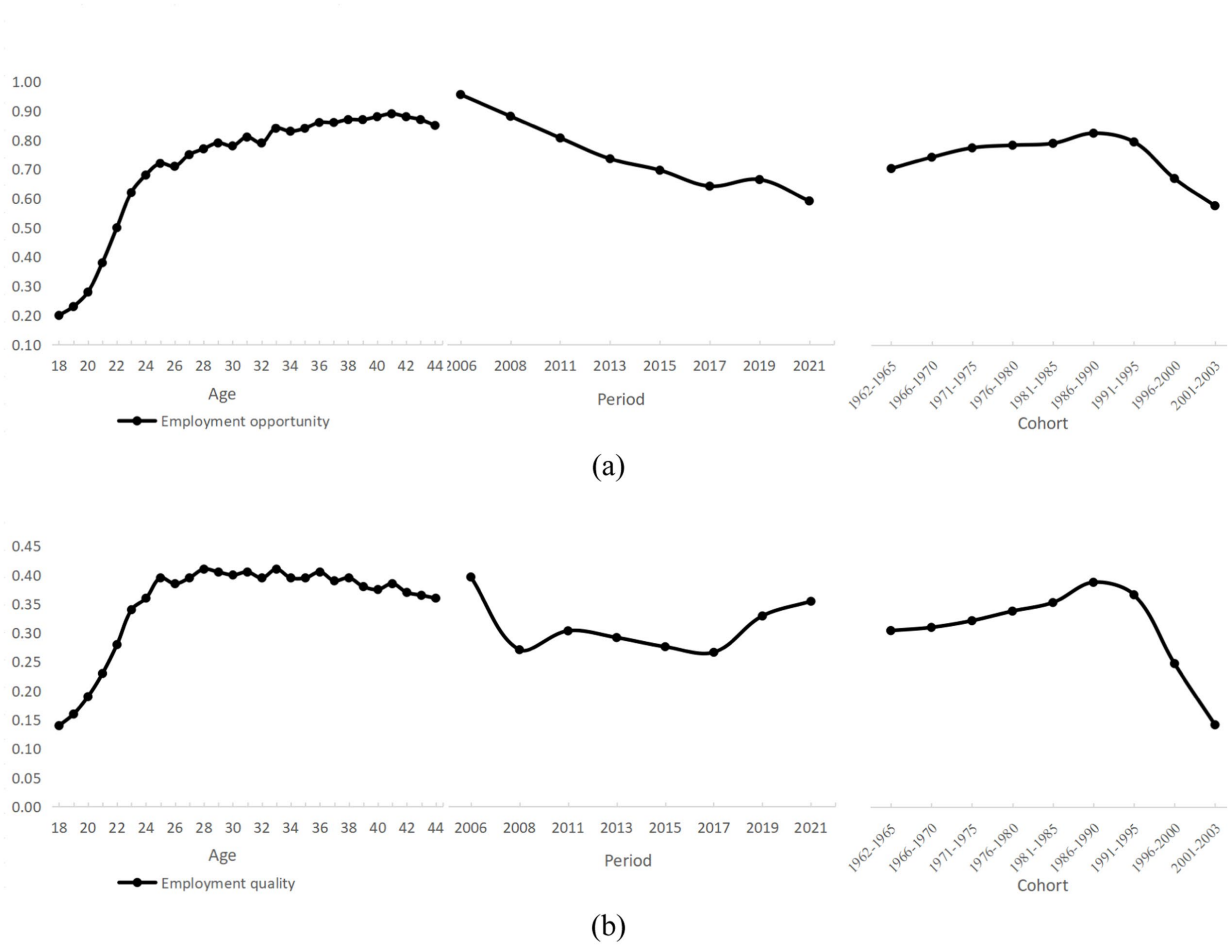
**(a)** Trends in employment opportunities for youth by age, period and cohort; **(b)** Trends in employment quality for youth by age, period, and cohort.

Table 2 presents the HAPC model results for youth employment opportunities and employment quality. Among them, models (1) and (3) serve as baseline models, including only four variables: age, age-squared, period, and cohort. Models (2) and (4) expand on these by incorporating control variables such as gender, marital status, household registration, and geographic location. The data indicate that age, period, and cohort all have a significant impact on both youth employment opportunities and employment quality. To further interpret these trends, the visual trends of the age effects on the employment status of Chinese youth are plotted, as shown in Figures 1a,b.

**Table 2:** Results of the HAPC model of youth employment status.

Variable	Employment opportunities		Employment quality	
	(1)	(2)	(3)	(4)
Intercept term	−1.245***	−1.412***	−0.562***	−0.660***
Age	0.114***	0.119***	0.057***	0.059***
Age squared	−0.002***	−0.002***	−0.001***	−0.001***
Gender		0.147***		0.060***
Marital status		−0.028***		−0.033***
Household registration (Ref: Rrural)				
Non-farmer		0.008		0.125***
Urban		0.005		0.099***
Political affiliation		0.097***		0.099***
Region (Ref: Eastern)				
Central		−0.031***		−0.049***
Western		0.006		−0.041***
Period	0.381***	0.364***	0.184***	0.166***
Cohort	0.306***	0.306***	0.206***	0.209***
N	27,958	27,958	27,958	27,958
AIC	25,665	24,366	−4,822	−9,101
BIC	25,715	24,473	−4,773	−8,994

*p < 0.05, **p < 0.01, ***p < 0.001.

The results show that both employment opportunities and job quality begin to rise from age 18, reflecting the school-to-work transition and the rapid accumulation of early labor market experience. Employment opportunities increase steadily throughout youth, peaking around age 40—an upward trajectory spanning nearly the entire youth stage. By contrast, employment quality climbs sharply before age 25, after which it enters a prolonged plateau. In other words, the attainment of high-quality job attributes—such as stability, income, and working conditions—tends to level off earlier than the probability of securing employment.

The period effects for employment opportunities and employment quality diverged between 2006 and 2021. Youth employment opportunities displayed a persistent downward trend, indicating increasingly fierce competition in the labor market. The changes in employment quality were more complex, moving through three distinct phases: a sharp decline, followed by low-level fluctuation, and finally gradual recovery. Similar conclusions emerge from Wang’s (2025) analysis using data from the China Family Panel Studies (CFPS) (2016–2022). A likely explanation lies in the technological transformation of industries: while innovation has created more high-quality jobs, it has simultaneously displaced many traditional roles, fostering a three-tiered labor market marked by high-skill premiums, mid-skill polarization, and low-skill substitution.

Regarding cohort effects, the divergence between employment opportunities and job quality is relatively modest, with both exhibiting an overall pattern of slow increase followed by sharp decline. Notably, both indicators remain relatively low for the “post-60s” and “post-00s” cohorts, whereas the “post-85 s” cohort achieves consistently higher values—suggesting that this generation has benefited from a more favorable economic and labor market environment. These patterns may reflect a confluence of factors, including the pace of economic development, industrial restructuring, rising educational attainment, and shifts in labor demand.

### 4.3 HAPC model analysis of the impact of higher education on employment status

Table 3 presents the HAPC model results examining the influence of higher education on youth employment status. Models (5)–(8) focus on employment opportunities as the dependent variable, while models (9)–(12) analyze employment quality. Specifically, models (5) and (9) serve as baseline models, incorporating only higher education, age, age squared, and control variables. The findings indicate that higher education positively affects both employment opportunities and employment quality, suggesting that individuals with higher education credentials are more likely to secure employment and attain better job quality than those without higher education.

**Table 3:** Results of the HAPC model of higher education on its employment status.

Variable	Employment opportunities				Employment quality			
	(5)	(6)	(7)	(8)	(9)	(10)	(11)	(12)
	Base model	Age effects	Period effects	Cohort effects	Base model	Age effects	Period effects	Cohort effects
Layer 1 variable								
Higher education	0.054***	−0.912***	0.046*	0.026	0.132***	−0.273***	0.131***	0.113***
Age	0.116***	0.091***	0.087***	0.095***	0.052***	0.034***	0.034***	0.039***
Age squared	−0.002***	−0.001***	−0.001***	−0.001***	−0.001***	0.000***	−0.000***	−0.000***
Higher education*Age/Higher education*Age^2^								
Higher education*Age		0.121***				0.086***		
Higher education*Age^2^		−0.002***				−0.001***		
Gender	0.147***	0.143***	0.148***	0.145***	0.062***	0.059***	0.063***	0.060***
Marital status	−0.019**	−0.035***	−0.020**	−0.030***	−0.01*	−0.023***	−0.009*	−0.019***
Household registration (Ref: Rural)								
Non-farmer	−0.01	−0.025***	−0.009	−0.023***	0.080***	0.069***	0.079***	0.069***
Urban	−0.007	−0.019*	−0.002	−0.016	0.071***	0.061***	0.072***	0.062***
Political affiliation	0.077***	0.055***	0.079***	0.066***	0.050***	0.033***	0.050***	0.038***
Region (Ref: Eastern)								
Central	−0.029***	−0.023***	−0.030***	−0.026***	−0.045***	−0.041***	−0.045***	−0.042***
Western	0.008	0.009	0.006	0.007	−0.036***	−0.035***	−0.036***	−0.035***
Layer2 random effects variance								
Period								
Higher education			0.342***				0.167***	
Intercept term	0.371***	0.362***	0.189***	0.354***	0.181***	0.179***	0.205***	0.198***
Cohort								
Higher education				0.267***				0.253***
Intercept term	0.307***	0.290***	0.289***	0.253***	0.214***	0.190***	0.244***	0.201***
N	27,958	27,958	27,958	27,958	27,958	27,958	27,958	27,958
AIC	24,298	23,779	24,532	24,327	−10,706	−11,751	−10,658	−11,252
BIC	24,413	23,911	24,655	24,451	−10,590	−11,619	−10,535	−11,128

*p < 0.05, **p < 0.01, ***p < 0.001.

#### 4.3.1 Age effects

**Figure 2:**
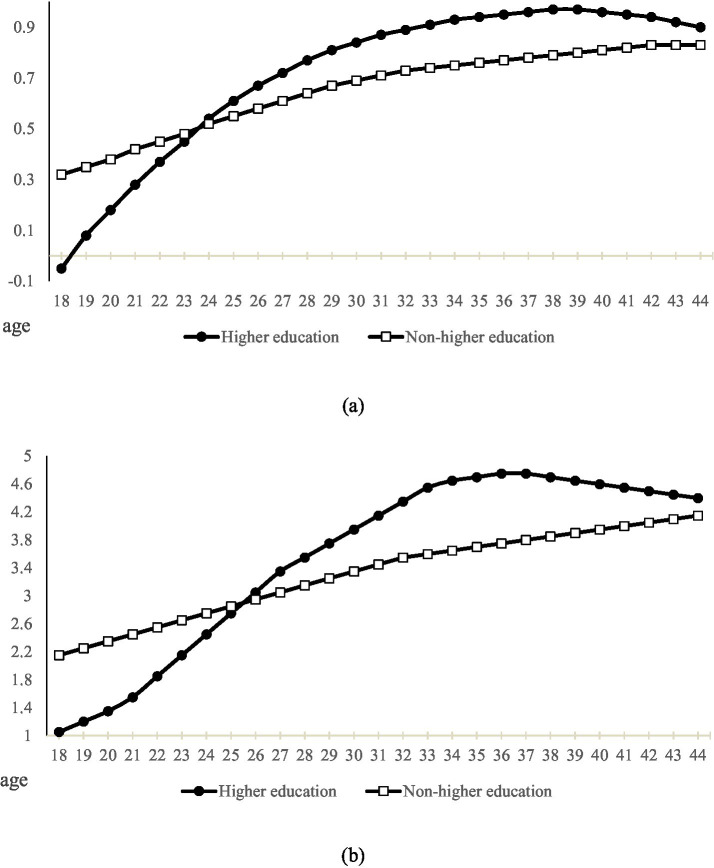
**(a)** Trends in age variation in employment opportunities for youth with different levels of education. **(b)** Trends in age variation in employment quality for youth with different levels of education.

Models (6) and (10) further investigate the age effects. The regression results indicate that the interaction term between higher education and age is significantly positive (β = 0.121, *p* < 0.001; β = 0.086, p < 0.001), whereas the interaction term between higher education and age squared is significantly negative (β = −0.002, p < 0.001; β = −0.001, p < 0.001). These patterns indicate a nonlinear relationship between higher education and both youth employment opportunities and employment quality. To visualize these model-based findings, Figures 2a,b present the predicted curves for returns to higher education—compared with non-higher-education counterparts—across different age groups.

Figure 2a displays an inverted U-shaped pattern in the employment opportunity returns to higher education across age groups. Before the age of 24, individuals with higher education enjoy fewer employment opportunities than their counterparts without higher education. Thereafter, the net effect for the higher-education cohort rises sharply, overtaking that of the non-higher-education group. This upward trend persists until the inflection point at age 39, after which the gap between the two groups gradually narrows. Figure 2b presents a similar inverted U-shaped trajectory for employment quality among higher education graduates. Between ages 18 and 25, their employment quality remains lower than that of non-graduates. From ages 25 to 37, it increases steadily, reaching a peak at age 37. Beyond this age, returns to employment quality for higher education graduates begin to decline. By contrast, the employment quality of non-graduates improves at a slower but more consistent pace throughout the same age span.

#### 4.3.2 Period effects

**Figure 3:**
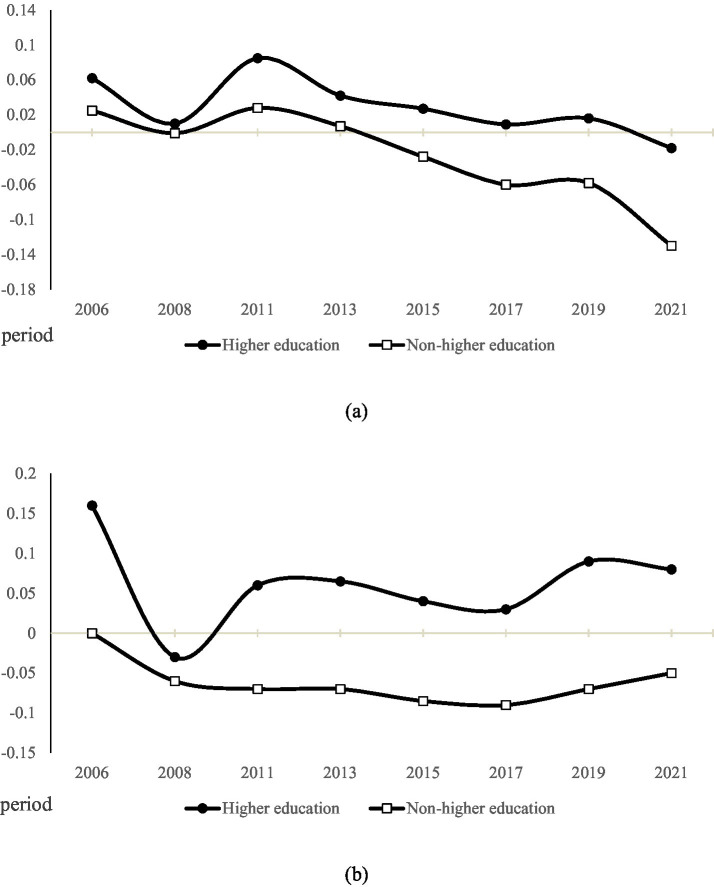
**(a)** Trends in period variation in employment opportunities for youth with different levels of education. **(b)** Trends in period variation in employment quality for youth with different levels of education.

Models (7) and (11) examine the period effects, focusing on how shifts in the macroeconomic environment reshape the relationship between youth higher education and employment outcomes. The regression results show that the period coefficients are significant for both employment opportunities and employment quality (β = 0.342, *p* < 0.001; β = 0.167, p < 0.001). These findings suggest that returns to higher education—in terms of both employment opportunities and job quality—vary substantially across survey periods. To visualize these patterns, we plotted the predicted curves for both indicators among youth with higher education across different periods, as shown in Figures 3a,b.

Figure 3a depicts the trend in employment opportunities for the higher-education and non-higher-education cohorts after controlling for age and birth cohort. Overall, the higher-education cohort consistently maintained a higher period effect than the non-higher-education cohort. Both groups reached relatively high levels in 2011, followed by a gradual decline. However, this decline was far steeper for the non-higher-education group, with the negative gap widening steadily after 2015 and hitting its lowest point in 2021.

Figure 3b illustrates the trajectory of employment quality returns across periods. The higher-education cohort started from a particularly high level in 2006 but experienced a sharp drop in 2008. From 2011 onward, employment quality began to recover, reaching the second-highest value of the entire period in 2019. In contrast, the non-higher-education cohort exhibited far slower changes. Its employment quality declined in 2008 and then remained in a prolonged low-level plateau, with only minor rebounds in 2019 and 2021.

#### 4.3.3 Cohort effects

**Figure 4:**
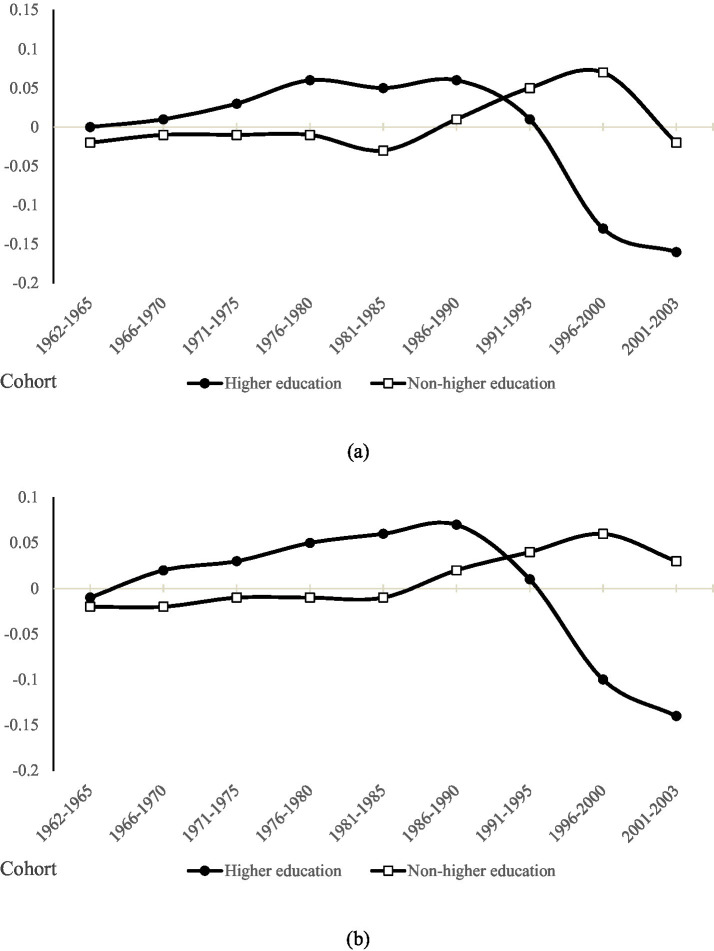
**(a)** Trends in cohort variation in employment opportunities for youth with different levels of education. **(b)** Trends in cohort variation in employment quality for youth with different levels of education.

Model (8) and Model (12) focus on how cohort differences affect the relationship between higher education and employment opportunities and employment quality among young people. The regression results show highly significant coefficients for both variables (β = 0.267, *p* < 0.001; β = 0.253, p < 0.001), indicating that birth cohort exerts a substantial influence on the returns to higher education. Figures 4a,b visualize these patterns.

The charts reveal that cohort-specific trends in higher education’s influence on employment opportunities and job quality are broadly aligned. Among the higher-education group, both indicators increased steadily across earlier birth cohorts, peaking with the “1986–1990” generation. After this cohort, the effect declined sharply and even turned significantly negative for certain later cohorts. By contrast, cohort effects for the non-higher-education group changed only marginally between the “1962–1965” and “1981–1985” generations. They then rose to a peak in the “1991–1995” generation and, although declining thereafter, remained above the levels observed for the higher-education group. In short, the relevance of higher education to employment opportunities and quality shows a “gentle rise—sharp decline” trend, while the relevance of non-higher education is characterized by “gentle fluctuation—slight rise.”

Table 4 presents the findings regarding the moderating mechanism of higher education expansion on its impact on employment opportunities and employment quality. The results indicate that educational expansion has a significant negative moderating effect on both the cohort effects related to employment opportunities and employment quality. This highlights the complex and dual nature of higher education expansion: while it offers upward mobility for youth groups, it also creates congestion and competition effects (Zhuo, 2021). Specifically, an oversupply of graduates reduces individuals’ competitive advantage, diminishes the scarcity value of education, and leads to a gradual decline in the marginal returns of employment opportunities. This underscores the importance of balancing the scale of higher education expansion with structural optimization in education policy to ensure the long-term sustainability of intergenerational human capital accumulation.

**Table 4:** Results of the analysis of the moderating effect.

Variable	Employment opportunity		Employment quality	
	(13)	(14)	(15)	(16)
Higher education	0.100***	0.099***	0.118***	0.118***
Educational expansion	−0.055***	−0.031	−0.023***	−0.009
Interaction term				
Higher education × educational expansion		−0.068***		−0.048***
Control variable	---	---	---	---
N	51,418	51,418	51,418	51,418
AIC	58,023	58,021	−13,286	−13,290
BIC	58,165	58,173	−13,143	−13,138

--- indicates controlled; *p < 0.05, **p < 0.01, ***p < 0.001.

### 4.4 Robustness tests

**Figure 5:**
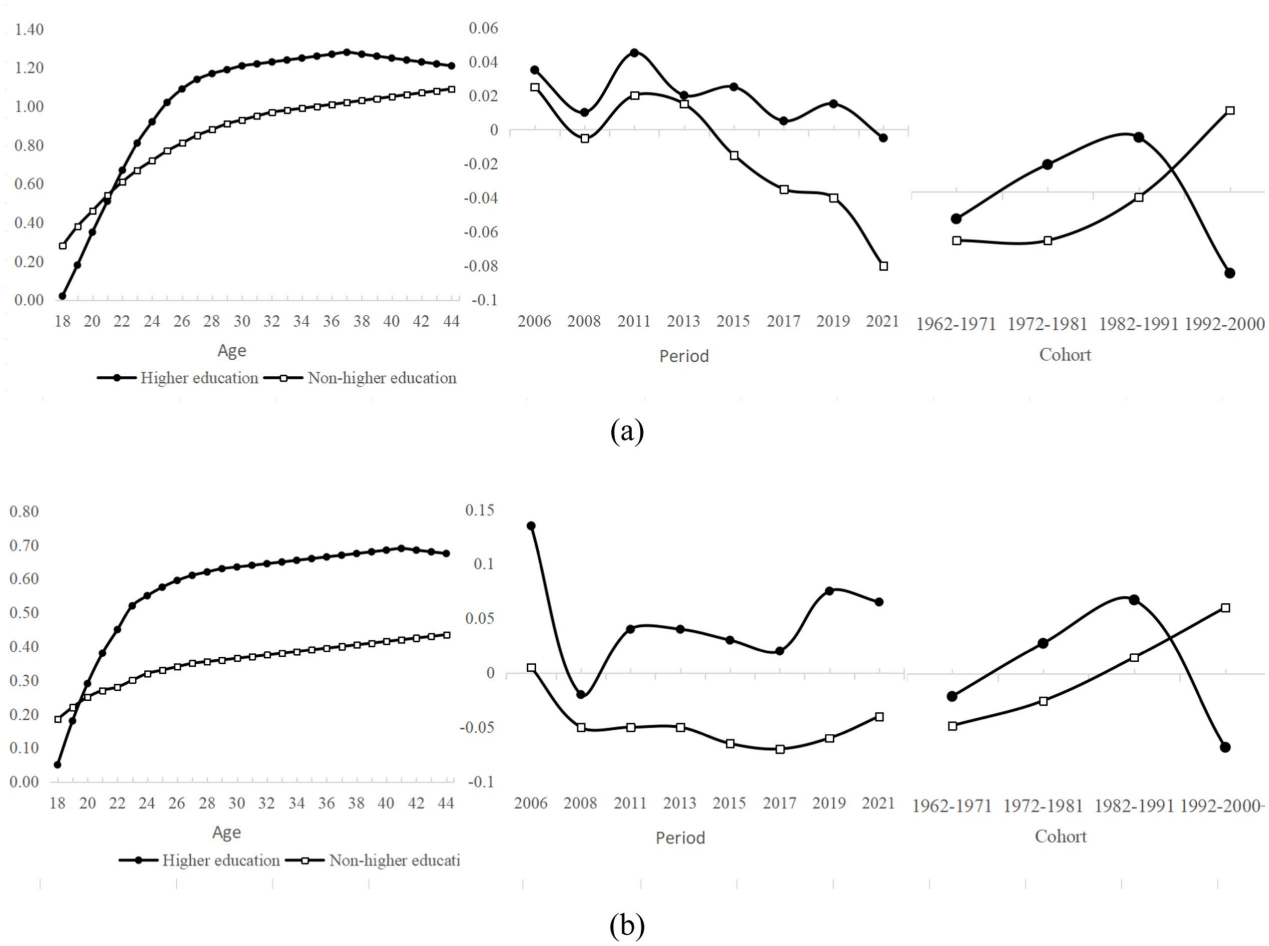
**(a)** Trends in employment opportunities for youth by age, period and cohort (10 years as a cohort). **(b)** Trends in employment quality for youth by age, period, and cohort (10 years as a cohort).

This study employed two approaches to assess the robustness of the empirical findings. First, in terms of variable treatment, we followed the approach of some scholars by grouping cohorts into 10-year intervals instead of the 5-year intervals used previously, and subsequently applied these groups to the multilayer cross-sectional random effects model (Liang, 2007; Zeng and Zheng, 2016). Second, a dummy variable grouping model was utilized in the estimation process, where age, period, and cohort were included as dummy variables. The period was represented by the actual survey year, while age and cohort were grouped into 5-year intervals (Wu and Li, 2021). The test results indicate that both methods yielded conclusions consistent with those of the HAPC model. As a result, we can conclude that the empirical findings are both robust and reliable. Due to space constraints, only the results from method I are shown, as illustrated in Figures 5Aa,b.

## 5 Discussion

### 5.1 Age effect

In China, individuals typically complete their higher education between the ages of 22 and 24. Consequently, most young people under the age of 24 are either still in university or have just entered the labor market, which results in relatively limited employment opportunities for this age group. However, once they graduate, the range of employment options expands considerably, particularly in fields where higher education is in high demand, such as technology, management, and scientific research, where they often have a competitive advantage. This educational advantage will continue until the age of 38, and then slowly decline, eventually approaching the non-tertiary education at the age of 44. This pattern mirrors findings from a study by scholar Li on China’s labor market between 2000 and 2015, where the “inverted U-shape” phenomenon was notably more pronounced in groups with higher education levels (Li and Wu, 2025). There are two main explanations for this trend. First, after accumulating economic and social capital, older workers often experience greater life stability, reducing their need to continue working. As a result, many choose to retire voluntarily and enjoy their post-career life. Second, older workers are more vulnerable to the challenges posed by knowledge obsolescence, rigid retirement policies, and age discrimination, making them more likely to exit the labor market involuntarily compared to their younger, highly educated counterparts (Yue, 2017; Wei et al., 2024).

In a highly competitive job market, individuals with higher education typically have a competitive edge over those without it, often securing higher-quality positions in industries that offer higher salaries, better social security, more comfortable working conditions, and greater career development opportunities. As a result, they generally rate their employment quality higher. However, this advantage in employment quality driven by education is not guaranteed to last indefinitely. After surpassing the prime career development period (around the age of 37), age-related disadvantages begin to emerge and gradually diminish the benefits of quality treatment associated with higher education. This phenomenon is influenced by two main factors: first, at the individual level, as workers age, the imbalance between their health and job intensity increases, while the amount of non-market labor spent on family care and elderly support rises. This leads to the need for workers to either voluntarily downgrade their employment quality or exit their core positions earlier. Second, in the broader external environment, Chinese companies often implement a “35-year-old employment threshold,” indirectly excluding middle-aged candidates and promotions through criteria like “team rejuvenation” and “innovation and vitality.” This contributes to a decline in employment quality for highly educated workers as they age (Kivinen and Rinne, 1993).

### 5.2 Period effects

In 2006, the higher education cohort maintained a clear advantage in employment opportunities. However, this advantage declined in 2008, likely due to the global financial crisis. Following the launch of China’s “4 trillion yuan” economic stimulus package and targeted employment support measures—such as the Notice on Doing a Good Job in Promoting Employment and the Interim Provisions on Employment Work for Graduates of Regular Higher Education Institutions—their employment advantage rebounded by around 2011 and reached a temporary peak. This pattern illustrates that policy interventions can effectively mitigate the employment pressures faced by highly educated groups during economic downturns. Since 2011, however, two cumulative forces have eroded this advantage: a slowdown in economic growth (per capita GDP growth decreased from 8.9 to 7.1% in 2011) and the large-scale expansion of higher education since 1999. The number of graduates increased by 64.7% between 2006 and 2012, reaching 6.8 million, and further rose to 9.09 million by 2021. These trends have contributed to a sustained decline in the relative employment advantage of higher education graduates. Although China’s industrial restructuring since 2014 has spurred rapid growth in high value-added sectors such as services and technological innovation, job creation has lagged behind the influx of university graduates into the labor market, intensifying competition for positions. At the same time, “degree inflation” has heightened competitive pressure on workers with lower educational attainment, especially since 2015. This phenomenon is closely tied to the Fourth Industrial Revolution, in which the widespread adoption of technologies such as the internet, big data, and artificial intelligence has created high-skill jobs while accelerating the elimination of many low-skill functions. As a result, non-college graduates now face the dual challenge of reduced demand for low-skill work and intensified competition from degree holders.

Similarly, the global financial crisis caused a temporary decline in the employment quality of the higher education cohort. During the subsequent economic recovery, two main factors drove its rebound. First, the share of labor income remained relatively stable, as profit growth from the recovering economy supported workers’ compensation. Second, institutional safeguards were strengthened through measures such as the Labor Contract Law (2008) and the Social Insurance Law (2009), which significantly improved job stability and social security. In addition, targeted government policies for college graduates—such as expanded employment subsidies and entrepreneurship programs—collectively fostered a more favorable employment environment for this group. In sharp contrast, the employment quality of non-college graduates stayed negative throughout the observation period, with the decline continuing until 2017. This trend is consistent with labor market segmentation theory: lower-educated workers are concentrated in the “secondary labor market,” characterized by low wages, insecure jobs, and limited career mobility. As the economy shifted toward high-skill, technology-intensive industries, their relative position steadily worsened. Although there was a modest rebound after 2017—driven by service sector growth in areas such as e-commerce logistics—their employment quality remained far below that of their highly educated counterparts. Notably, during the COVID-19 pandemic, the employment quality of the higher education cohort did not fall significantly and stayed at a relatively high level. This resilience is largely explained by their concentration in robust sectors such as information technology and online services, along with the rapid adoption of flexible work arrangements like remote work. By contrast, low-skilled jobs dependent on physical presence were hit hard, worsening the situation for the non-higher education group.

### 5.3 Cohort effects

According to Mannheim’s theory of “socialization,” major historical events occurring during formative developmental periods can profoundly influence a cohort’s values, attitudes, and behaviors, setting them apart from previous generations. Such experiences create a shared identity and a distinct social character unique to that cohort. In China, individuals born between the 1960s and 1990s grew up during the country’s golden era of reform, opening-up, and rapid economic expansion. Their upward trajectory in both employment opportunities and job quality was therefore unsurprising. Several factors contributed to this trend. First, the resumption of the national college entrance examination in 1977 allowed talented young people from across the nation to access systematic higher education, making academic credentials a critical gateway to career advancement. During the planned economy, university graduates enjoyed the “guaranteed job placement” policy, which ensured stable employment and higher social status, thereby cementing higher education as a key source of occupational security. Second, the post-reform transition toward a market economy spurred industrial development in technology- and knowledge-intensive sectors, generating strong demand for highly educated talent and amplifying the “skill premium” effect of education. In the 1990s, the emergence of the internet era further elevated the role of this educated cohort, positioning them at the forefront of technological innovation and industrial transformation. As a result, compared with other groups, higher education graduates were more likely to secure better pay through work in knowledge-, technology-, and skill-intensive fields, leading to a steady improvement in their employment quality over time.

For post-90s workers and later generations, the job market landscape has undergone significant changes. First, with the transition to a market economy, the government introduced the policy of “independent employment for workers, market-regulated employment, and government-supported employment.” As a result, university graduates no longer benefit from the institutional guarantee of “uniform job allocation” and must rely entirely on their own abilities and market competitiveness to secure employment, leading to heightened uncertainty in the job search process. Second, the expansion of higher education has diminished the exclusivity of a bachelor’s degree, transforming it from a symbol of “elite status” into a form of “basic education.” Consequently, some industries have raised their entry requirements from a bachelor’s to a master’s or doctoral degree, gradually reducing the overall returns of higher education. Consistent with the findings of the moderating effect test in this study, some scholars analyzing the job-seeking experiences of Korean college graduates through qualitative research have observed that higher education expansion intensifies the “vertical stratification” of universities, exacerbating employment inequality among graduates (Li and Zheng, 2022). However, it is important to recognize that the expansion of higher education is not the sole factor contributing to employment challenges. Labor market segmentation, incomplete employment information (Lai and Tian, 2005), socio-economic conditions (Cui, 2011), and individual limitations in skills (Yue, 2017) and social capital all play a role in graduates’ job search difficulties (Yue, 2023). Additionally, there has been a shift in young people’s employment perceptions. Increasingly, young individuals are moving away from the pursuit of traditional “stable jobs” and are instead opting for freelancing, internet entrepreneurship, self-media operations, and other emerging career paths. Success in these fields is more dependent on personal skills and innovation than on academic credentials, contributing to the decline in the returns of higher education in certain job markets.

### 5.4 Policy recommendations

Differentiated policy interventions should be tailored to the needs of different age groups, ranging from individual capacity building to the strengthening of social support systems. First, individual capacity building. (1) Strengthening early career preparation: For individuals aged 18–24, improve the transition from higher education to the workplace through internships, project-based learning, and vocational skills training. These measures can shorten graduates’ adaptation period, enhance their initial employability, and improve the quality of their first jobs. (2) Extending the peak career period: For those in the prime career stage (ages 24–38), encourage enterprises to offer continuing education, cross-disciplinary training, and international exchange opportunities. This will help young professionals maximize their human capital potential while adapting to technological advancements and shifting job requirements. (3) Establishing a lifelong retraining system: For individuals aged 38 and above, whose returns to education may begin to decline, develop structured retraining programs that support career transitions and job redevelopment, thereby sustaining employability. Second, strengthening the social support system. (1) Building industry–education integration platforms: Led by local governments and involving universities, enterprises, and industry associations, establish cross-organizational talent development and internship bases to ensure a smooth transition from campus to the workplace. (2) Improving career guidance services: Formulate targeted youth employment promotion plans with clear objectives by age, industry, and region. Refine legislation to guarantee fairness in recruitment, promotion, and compensation, and establish youth employment monitoring and early-warning systems to track market dynamics in real time. (3) Accelerating economic transformation to create high-quality jobs: Increase state investment in innovative enterprises within emerging industries, support the incubation of internationally competitive “specialized, refined, distinctive, and novel” small and medium-sized enterprises (SMEs), and expand the supply of high-quality jobs while improving the overall quality and efficiency of economic development.

Comprehensive safeguards should be implemented across all stages, ranging from macroeconomic regulation to reforms in educational supply. First, macroeconomic regulation. (1) Countercyclical employment policies: During periods of economic downturn or industrial contraction, expand job creation in public services, infrastructure projects, and high-tech industries, with priority given to young job seekers. (2) Industry-specific support: In sectors severely affected—such as services and foreign trade—provide subsidies for skill transitions and startup funding to highly educated youth, helping maintain both the scale and quality of employment. (3) Promoting regional balance: Use tax incentives and industrial development policies to attract high-quality enterprises to central and western regions suffering from talent outflow, thereby increasing youth employment opportunities. Second, educational supply reform. (1) Dynamic adjustment of disciplinary structures: Align university program structures with evolving industrial needs by expanding enrollment in emerging fields—such as the digital economy, new energy, and healthcare—while reducing programs misaligned with market demand. (2) Strengthening practical education: Facilitate collaboration between universities and industry to co-develop curricula that integrate real-world projects and industry challenges, thereby enhancing students’ complex problem-solving skills. (3) Multidimensional employment quality evaluation: Establish an evaluation system in which governments, universities, and third-party institutions jointly publish annual graduate employment quality reports, providing reliable data to guide educational policy and inform student career choices.

Tailored support measures should be implemented for different age groups, ranging from compensating cohorts with declining returns to empowering younger generations. First, enhancing the competitiveness of the declining-returns cohort. (1) Capacity Compensation Program: For individuals born after 1990 who face diminishing returns on education, provide free or subsidized advanced skills training and digital competency enhancement programs to help them overcome employment ceilings. (2) Strengthening resource allocation: Promote deep urban–rural integration by improving rural living environments, boosting agricultural productivity, and encouraging young people with rural household registrations to return to their hometowns for rural development. (3) Expanding overseas and cross-regional employment: Establish international internship exchange schemes and domestic job-sharing platforms in major cities to create diverse employment pathways for youth. Second, consolidating the advantages of the high-return cohort. (1) Innovating incentive mechanisms: Encourage highly educated professionals born between 1955 and 1990 who remain active in the workforce to engage in mentorship programs, lead innovation projects, and participate in entrepreneurial incubation, thereby strengthening their role as mentors for the younger generation. (2) Institutionalizing expertise: Convert the accumulated industry experience and technical know-how of these professionals into standardized training modules and case libraries to build a sustainable human capital development system. (3) Promoting intergenerational integration: Foster cross-generational collaborative projects, enhance information sharing, and promote joint innovation among different cohorts to increase labor productivity and improve overall employment quality.

### 5.5 Strengths and limitations

The marginal contributions of this study are threefold. First, by adopting a multidimensional temporal perspective to examine the impact of higher education on youth employment, this research integrates the fields of higher education, labor market dynamics, social change, and historical context. This comprehensive approach addresses the limitations of traditional cross-sectional and single-cohort longitudinal analyses, filling existing gaps in understanding both the dynamic evolution of youth employment and the shortage of in-depth contextual analysis. Second, the study clearly differentiates between the effects of higher education on two distinct dimensions—youth employment opportunities and employment quality—and further analyzes the moderating role of educational expansion in shaping the relationship between them. Finally, from a practical standpoint, the findings provide valuable policy insights, offering evidence-based recommendations for governments to design targeted and effective youth employment strategies.

Although this study strives for rigor, several limitations warrant attention in future research. First, the analysis does not fully capture the heterogeneity among youth cohorts across multiple temporal and demographic dimensions, such as gender, age group, region, and income level. Future studies should refine their research designs to better explore the distinct trajectories of different social groups with respect to educational returns and employment quality. Second, while multiple covariates were controlled for, certain potential confounding factors may remain unaccounted for. Expanding the range of control variables would help strengthen the robustness and generalizability of future findings. Third, as the results are derived from observational data and statistical models, they primarily reveal correlations rather than definitive causal relationships. To improve the validity and credibility of causal inferences, future research could integrate approaches such as natural experiments, instrumental variable techniques, or panel data fixed-effects models.

## 6 Conclusion

This study identifies the multidimensional patterns of youth employment opportunities and job quality across the life course, over time, and across generations. It shows that marked differences emerge between educational attainment groups under the combined influence of macroeconomic conditions and structural factors. The results reveal a clear age effect for employment opportunities: they rise steadily from age 18 throughout the youth stage, peaking at around age 40. In contrast, employment quality increases rapidly before age 25, after which it remains in a prolonged phase of high-level fluctuation. As time progressed, employment opportunities for both higher education and non-higher education groups have generally shown a fluctuating decline since 2006. However, the decline in higher education has been relatively slower and has consistently remained above that of the non-higher education group. As birth cohorts become younger, the influence of higher education on both employment opportunities and job quality follows a “gradual rise–sharp decline” trajectory, with the turning point occurring among those born between 1986 and 1990. By contrast, for non-higher education graduates, the pattern is one of “moderate fluctuation–slight increase,” with an earlier turning point among those born between 1981 and 1985.

Overall, although the returns to higher education diminish in the later stages across all three temporal dimensions, the faster deterioration in employment opportunities and job quality among non-college graduates has maintained the relative advantage of college graduates in the labor market. This advantage may even strengthen further during specific periods.

## Data Availability

The datasets presented in this study can be found in online repositories. The names of the repository/repositories and accession number(s) can be found at: Chinese Academy of Social Sciences for providing the Chinese Social Study (CSS) data (https://css.cssn.cn/css_sy/).
